# P Wave Dispersion and Maximum P Wave Duration Are Associated with Renal Outcomes in Chronic Kidney Disease

**DOI:** 10.1371/journal.pone.0101962

**Published:** 2014-07-09

**Authors:** Jiun-Chi Huang, Shu-Yi Wei, Szu-Chia Chen, Jer-Ming Chang, Chi-Chih Hung, Ho-Ming Su, Shang-Jyh Hwang, Hung-Chun Chen

**Affiliations:** 1 Division of Nephrology, Department of Internal Medicine, Kaohsiung Medical University Hospital, Kaohsiung Medical University, Kaohsiung, Taiwan; 2 Division of Cardiology, Department of Internal Medicine, Kaohsiung Medical University Hospital, Kaohsiung Medical University, Kaohsiung, Taiwan; 3 Department of Internal Medicine, Kaohsiung Municipal Hsiao-Kang Hospital, Kaohsiung Medical University, Kaohsiung, Taiwan; 4 Faculty of Medicine, College of Medicine, Kaohsiung Medical University, Kaohsiung, Taiwan; 5 Faculty of Renal Care, College of Medicine, Kaohsiung Medical University, Kaohsiung, Taiwan; 6 Department of Internal Medicine, Kaohsiung Municipal United Hospital, Kaohsiung, Taiwan; 7 Yuh-Ing Junior College of Health Care & Management, Kaohsiung, Taiwan; University Medical Center Groningen and University of Groningen, Netherlands

## Abstract

P wave parameters measured by 12-lead electrocardiogram (ECG) are commonly used as a noninvasive tool to evaluate left atrial enlargement. This study was designed to assess whether P wave parameters were associated with renal outcomes in chronic kidney disease (CKD) patients. This longitudinal study enrolled 439 patients with CKD stages 3–5. Renal end points were defined as the commencement of dialysis or death. Change in renal function was measured using the estimated glomerular filtration rate (eGFR) slope. We measured two ECG P wave parameters corrected for heart rate, i.e., corrected P wave dispersion and corrected maximum P wave duration. The values of P wave dispersion and maximum P wave duration were 88.8±21.7 ms and 153.3±21.7 ms, respectively. During the follow-up period (mean, 25.2 months), 95 patients (21.6%) started hemodialysis and 30 deaths (6.8%) were recorded. Multivariate Cox regression analysis identified that increased P wave dispersion [hazard ratio (HR), 1.020; 95% confidence interval (CI), 1.009–1.032; *P*<0.001] and maximum P wave duration (HR, 1.013; 95% CI, 1.003–1.024; *P* = 0.012) were associated with progression to renal end points. Furthermore, increased P wave dispersion (unstandardized coefficient β = –0.016; *P* = 0.037) and maximum P wave duration (unstandardized coefficient β = –0.014; *P* = 0.040) were negatively associated with the eGFR slope. We demonstrated that increased P wave dispersion and maximum P wave duration were associated with progression to the renal end points of dialysis or death and faster renal function decline in CKD patients. Screening CKD patients on the basis of P wave dispersion and maximum P wave duration may help identify patients at high risk for worse renal outcomes.

## Introduction

Both traditional risk factors, such as hypertension, diabetes, and dyslipidemia, and non-traditional risk factors, such as cardiovascular disease contribute to the risk of progressive renal function loss [1], [2]. Faster renal function decline has been significantly associated with high cardiovascular morbidity and mortality in patients with chronic kidney disease (CKD) [3], [4]. It is therefore essential to identify those CKD patients with faster renal function decline in order to initiate aggressive treatment interventions and improve survival.

Echocardiographic measures of left ventricular function and structure as well as left atrial size have been reported to predict adverse renal outcomes [5]–[7]. This implies that patients with cardiac dysfunction and left atrial enlargement might have faster renal function decline and adverse renal outcome. However, structural and functional abnormalities of the heart are frequently evaluated using echocardiography, which may preclude its application if echocardiography or experienced operators are not available. 12-lead electrocardiogram (ECG) is a simple, inexpensive, and noninvasive clinical tool to assess structural and functional abnormalities of the heart [8]. P wave parameters measured by 12-lead ECG have been reported to be useful tools for assessing the risk of left atrial enlargement, left ventricular hypertrophy (LVH), and left ventricular diastolic dysfunction [9]–[17]. Furthermore, P wave parameters have been independently associated with increased risk of both atrial fibrillation and recurrent transient ischemic attacks [18], [19]. However, there are limited studies evaluating whether P wave parameters are associated with progression to end-stage renal disease, decline in renal function, or death in CKD patients. The aim of this study in a cohort of patients with CKD stages 3–5 were to assess whether P wave parameters were associated with progression to the commencement of dialysis or death and whether these parameters were associated with the rate of renal function decline.

## Subjects and Methods

### Ethics statement

The study protocol was approved by the Institutional Review Board of the Kaohsiung Medical University Hospital (KMUH-IRB-20130305). Informed consent was obtained in written form from patients and all clinical investigations were conducted according to the principles of the Declaration of Helsinki. The patients gave consent for the publication of the clinical details.

### Study patients

The study was conducted in a regional hospital in southern Taiwan. From January 2007 to May 2010, we consecutively enrolled 505 pre-dialysis patients with CKD stages 3–5, according to the National Kidney Foundation-Kidney Disease Outcomes Quality Initiative (K/DOQI) guidelines [20] from our Outpatient Department of Internal Medicine. Patients with evidence of kidney damage lasting for more than 3 months were classified as CKD stages 3, 4, and 5, on the basis of an estimated glomerular filtration rate (eGFR) level (mL/min/1.73 m^2^) of 30–59, 15–29, and <15 respectively. Patients with atrial fibrillation, significant mitral valve disease, and inadequate ECG image visualization were excluded. Twenty-seven patients with less than three eGFR measurements during the follow-up period were excluded. Those patients who died (n = 9) or entered dialysis therapy (n = 30) within 3 months after enrollment were also excluded to avoid incomplete observation of changes in renal function. Finally, 439 patients (mean age, 66.0±12.0 years, 274 males) were included in this study.

### Assessment of P wave parameters

A standard 12-lead surface ECG (25-mm/s, 1-mV/cm, and 100-Hz) was performed in all study patients. An image analysis software system (Image Tool 3.0) was used to perform quantitative assessments. We assessed the correlation of two P wave parameters, corrected P wave dispersion and corrected maximum P wave duration, with progression to dialysis or death and decline in renal function. P wave dispersion was calculated as the difference between maximum and minimum P wave duration [21]. Both the P wave measurements were corrected for heart rate using Bazett’s formula, i.e., the corrected P wave parameters were equal to P wave parameters/(RR)^1/2^ [22].

### Definition of LVH

LVH was defined as Sokolow-Lyon voltage criteria via 12-lead ECG (sum of the amplitude of the S wave in lead V_1_ and the R wave in lead V_5_ or V_6_ greater than 3.5 mV) [23].

### Collection of demographic, medical, and laboratory data

Demographic and medical data including age, gender, smoking history (ever *versus* never), and comorbid conditions were obtained from medical records or patient interviews. The body mass index (BMI) was calculated as the ratio of weight in kilograms divided by square of height in meters. Laboratory data were measured from fasting blood samples using an autoanalyzer (Roche Diagnostics GmbH, D-68298 Mannheim COBAS Integra 400). Serum creatinine was measured by the compensated Jaffé (kinetic alkaline picrate) method in a Roche/Integra 400 Analyzer (Roche Diagnostics, Mannheim, Germany) using a calibrator traceable to isotope-dilution mass spectrometry [24]. The value of eGFR was calculated using the 4-variable equation in the Modification of Diet in Renal Disease (MDRD) study [25]. Dipsticks (Hema-Combistix, Bayer Diagnostics) were used to evaluate proteinuria. A dipstick test result of 1+ or more was defined as positive. Blood and urine samples were obtained within 1 month of study enrollment.

### Definition of renal end points

Renal end points were defined as the commencement of dialysis or death. In patients reaching study end points, renal function data were censored at the start of renal replacement therapy or death. The remaining patients were followed until March 2011. The commencement of dialysis was determined according to the regulations of the National Health Insurance for dialysis therapy based on laboratory data, nutritional status, and uremic symptoms and signs. Renal end points-free survival was defined as the time from study enrollment to the commencement of dialysis or death.

### Assessment of the rate of renal function decline

The rate of renal function decline was assessed by the eGFR slope, defined as the regression coefficient between eGFR and time in units of mL/min/1.73 m^2^/year. At least three eGFR measurements following 12-lead ECG examination were required to estimate the eGFR slope. Faster renal function decline was defined as a greater value of decline in the eGFR slope.

### Reproducibility

Thirty-five patients were randomly selected for evaluation of the interobserver variability of P wave dispersion and maximum P wave duration measurements by two independent observers. The same measurement was repeated twice, 1 week apart, to calculate the intraobserver variability. Mean percent error was calculated as the absolute difference divided by the average of the two observations.

### Statistical analysis

Statistical analysis was performed using SPSS version 17.0 (SPSS Inc., Chicago, IL, USA) for Windows. Data were expressed as percentages, mean ± standard deviation, mean ± standard error of the mean for the eGFR slope, or median (25^th^–75^th^ percentile) for triglycerides and number of serum creatinine measurements. The differences between groups were analyzed using the Chi-square test for categorical variables and using the independent *t*-test for continuous variables. Time to renal end points and covariates of risk factors were modeled using the Cox proportional hazards model. The survival curve for renal end points was derived using Kaplan–Meier analysis. Linear regression analysis was used to identify the factors associated with the eGFR slope. The association of P wave parameters with renal end points and the eGFR slope was assessed using a modified stepwise procedure in two modeling steps. The first model consisted of age and sex. In the second model, clinical and biochemical risk factors were added. A significant improvement in model prediction was found by calculating the improvement in –2 log likelihood or adjusted R square values. A difference was considered significant if the *P* value was less than 0.05.

## Results

Four hundred and thirty-nine non-dialyzed CKD patients were included. The mean age was 66.0±12.0 years and there were 274 males and 165 females. The average number of serum creatinine measurements during the follow-up period was eight (25^th^–75^th^ percentile: 6–12). The values of P wave dispersion and maximum P wave duration were 88.8±21.7 ms and 153.3±21.7 ms, respectively. The eGFR slope for all patients was –1.60±0.15 mL/min/1.73 m^2^/year. The underlying etiology of CKD in our patients was diabetic kidney disease in 232 (52.8%), non-diabetic glomerular diseases in 128 (29.2%), tubulointerstitial diseases in 46 (10.5%), hypertension in 23 (5.2%), and other diseases in 10 (2.3%). The clinical characteristics of patients with and without renal end points are shown in Table 1. Compared with patients without renal end points, patients with renal end points were found to have higher P wave dispersion, higher maximum P wave duration, higher prevalence of LVH, females outnumbered males, higher prevalence of diabetes mellitus (DM) and hypertension, higher systolic blood pressure, lower BMI, more advanced CKD stage, lower albumin, higher fasting glucose, lower hemoglobin, lower baseline eGFR, higher calcium-phosphorus product, higher uric acid, and higher prevalence of proteinuria.

**Table 1:** Comparison of baseline characteristics between patients with and without renal end points.

Characteristics	All patients(n = 439)	Patients without renalend points (n = 314)	Patients with renalend points (n = 125)	*P*
Electrocardiogram data				-
P wave dispersion (ms)	90 (76.5–100.8)	88.5 (73.7–97)	93.9 (84.1–105.9)	<0.001
Maximum P wave duration (ms)	154 (140.4–166.8)	151.5 (138–164)	157.3 (144.7–171.7)	0.002
LVH (%)	4.3	2.9	8.0	0.017
Age (year)	67.6 (57.1–76.3)	65.9 (56.9–76.2)	67.5 (57.6–76.4)	0.768
Male gender (%)	62.4	65.9	53.6	0.016
Smoking history (%)	31.0	30.6	32.0	0.770
Diabetes mellitus (%)	56.7	52.9	66.4	0.010
Hypertension (%)	82.2	79.0	90.4	0.005
Coronary artery disease (%)	11.6	10.8	13.6	0.413
Cerebrovascular disease (%)	15.3	14.0	18.7	0.249
Systolic BP (mmHg)	140 (129.5–153)	140 (126.5–150)	149 (130–163.8)	<0.001
Diastolic BP (mmHg)	80 (70–89)	80 (70–88)	79 (69–90)	0.397
Body mass index (kg/m^2^)	25.2 (22.8–27.5)	25.4 (23.3–27.7)	24.4 (22.1–26.9)	0.020
CKD stage				<0.001
Stage 3 (%)	38.7	51.0	8.0	
Stage 4 (%)	31.4	36.0	20.0	
Stage 5 (%)	29.8	13.1	72.0	
Laboratory parameters				
Albumin (g/dL)	4.1 (3.9–4.2)	4.1 (4.0–4.3)	3.8 (3.5–4.1)	<0.001
Fasting glucose (mg/dL)	107 (93–139)	104 (91–138)	114 (97–142.8)	0.024
Triglyceride (mg/dL)	138.5 (97–201)	139 (99–199.5)	135 (96–221.75)	0.449
Total cholesterol (mg/dL)	189 (162–220)	188 (162–219)	190.5 (160.8–230.3)	0.693
Hemoglobin (g/dL)	11.6 (9.8–13.3)	12.2 (10.7–13.7)	9.6 (8.5–10.9)	<0.001
Baseline eGFR (mL/min/1.73 m^2^)	25.2 (13.1–37.4)	30.4 (21.3–40.7)	11.1 (7.6–16.6)	<0.001
Calcium-phosphorus product (mg^2^/dL^2^)	37.4 (32.7–42.4)	36.2 (31.8–40.7)	41.9 (34.7–48.3)	<0.001
Uric acid (mg/dL)	7.9 (6.8–9.3)	7.8 (6.8–9.1)	8.4 (6.9–10.1)	0.001
Proteinuria (%)	65.2	55.1	90.4	<0.001

Abbreviations: LVH, left ventricular hypertrophy; BP, blood pressure; CKD, chronic kidney disease; eGFR, estimated glomerular filtration rate.

### Risk of progression to renal end points

The mean follow-up period was 25.2±12.7 months (range, 3.3–50.0 months). During the follow-up period, ninety-five patients (21.6%) started hemodialysis and 30 deaths were recorded in the 439 patients. Causes of death included fatal cardiovascular events (n = 13), infectious disease (n = 13), and other (n = 4). Among the 13 fatal cardiovascular events, 4 were myocardial infarction, 3 were pulseless electrical activity, 3 were ventricular fibrillation and 3 were heart failure. The multivariate Cox proportional hazards regression analyses of P wave dispersion and maximum P wave duration for renal end points are shown in Table 2. In the first analysis, P wave dispersion was associated with progression to renal end points in the multivariable model after adjustment for age and sex [hazard ratio (HR), 1.024; 95% confidence interval (CI), 1.014–1.034; *P*<0.001). The relationship remained significant after further adjustment for smoking, DM, hypertension, coronary artery disease, cerebrovascular disease, LVH, systolic and diastolic blood pressure, BMI, albumin, fasting glucose, log triglycerides, total cholesterol, hemoglobin, baseline eGFR, calcium-phosphorus product, uric acid, and proteinuria (HR, 1.020; 95% CI, 1.009–1.032; *P*<0.001). In the second analysis, there was also a significant prognostic value of maximum P wave duration in relation to renal end points (HR, 1.013; 95% CI, 1.003–1.024; *P* = 0.012) in multivariate analysis after adjustment for demographic, clinical, and biochemical factors. In addition, the commencement of dialysis and death were competitive risks for composite end points in this study. We also performed competing risk analysis and found that P wave dispersion (*P* = 0.046) remained significantly associated with progression to dialysis after multivariate analysis, but maximum P wave duration did not (*P* = 0.14).

**Table 2:** Predictors of progression to renal end points of dialysis or death using Cox proportional hazards model.

	P wave dispersion		Maximum P wave duration	
	HR (95% CI)	*P*	HR (95% CI)	*P*
Unadjusted	1.024 (1.014–1.034)	<0.001	1.016 (1.008–1.025)	<0.001
Age and sex adjusted	1.024 (1.014–1.034)	<0.001	1.017 (1.008–1.025)	<0.001
Multivariate adjusted	1.020 (1.009–1.032)	<0.001	1.013 (1.003–1.024)	0.012

Values express as hazard ratios (HR) and 95% confidence interval (CI).

Multivariate model: adjusted for age, sex, smoking history, diabetes mellitus, hypertension, and coronary artery disease, cerebrovascular disease, left ventricular hypertrophy, systolic and diastolic blood pressure, body mass index, albumin, fasting glucose, log triglycerides, total cholesterol, hemoglobin, baseline eGFR, calcium-phosphorus product, uric acid, and proteinuria.

**Figure 1:**
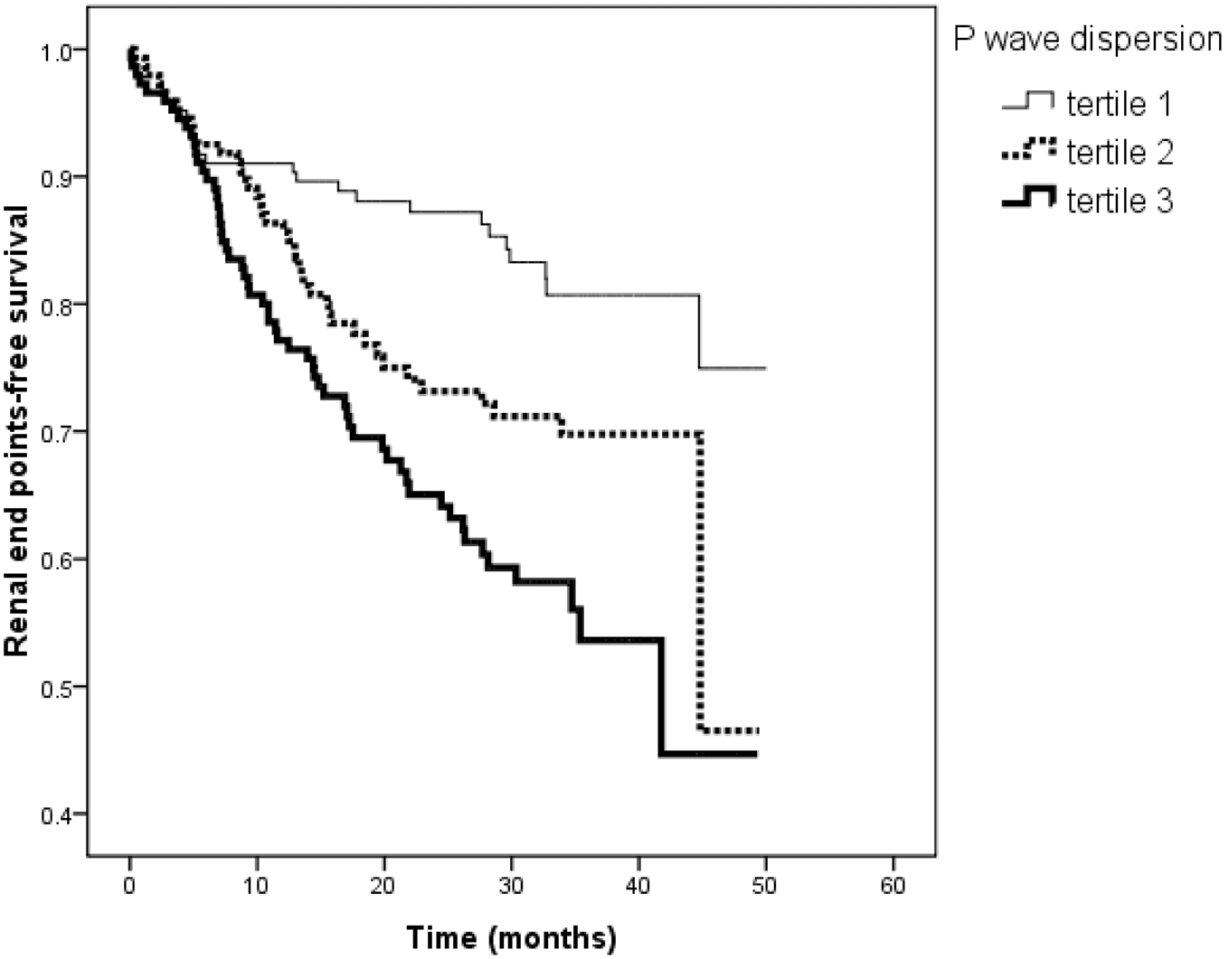
Kaplan-Meier analysis of renal end points-free survival according to the tertiles of P wave dispersion (log-rank *P*<0.001). The group with the highest two tertiles had a worse renal end points-free survival than that with the lowest tertile of P wave dispersion.

**Figure 2:**
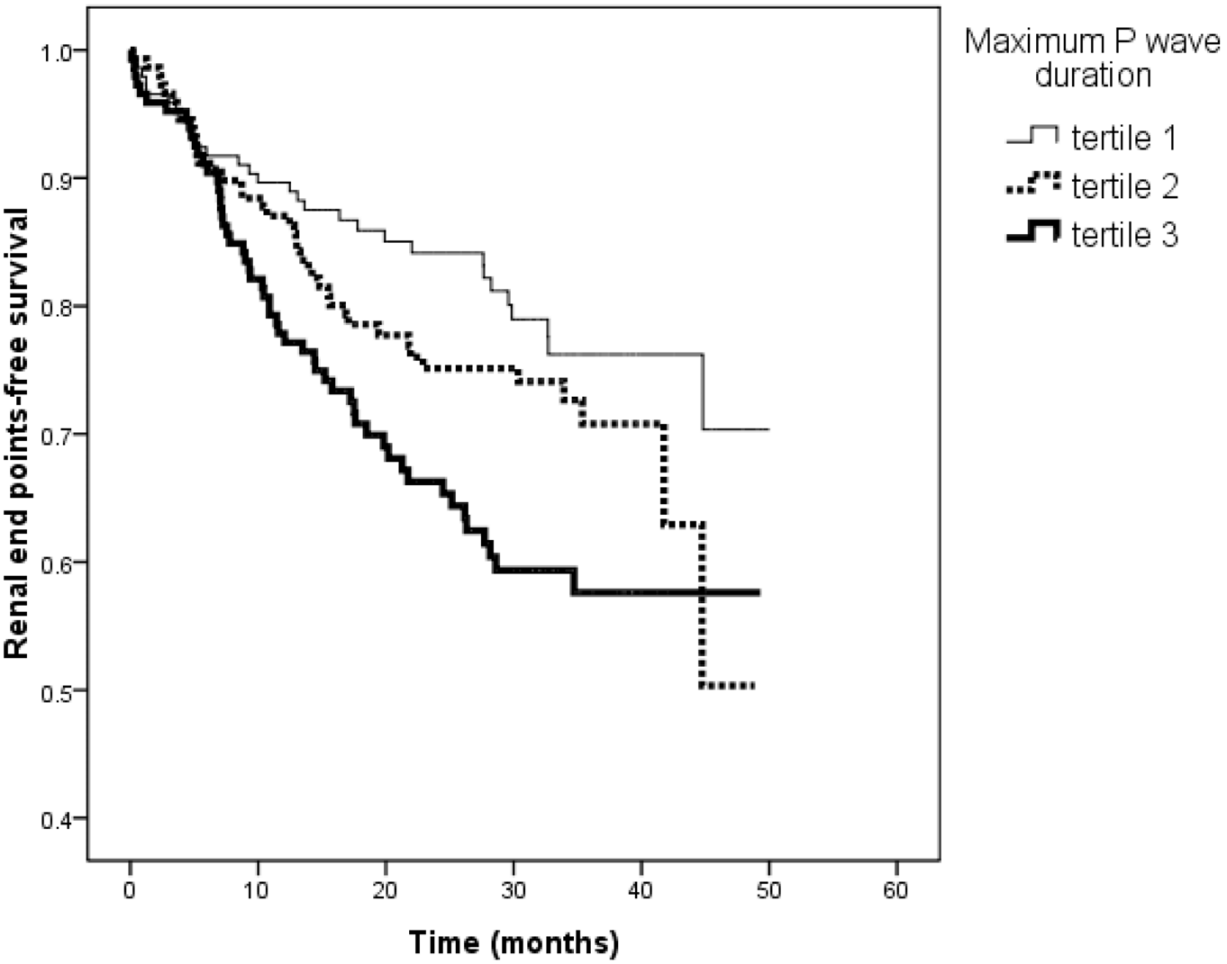
Kaplan-Meier analysis of renal end points-free survival according to the tertiles of maximum P wave duration (log-rank *P* = 0.003). The group with the highest tertile had a worse renal end points-free survival than that with the lowest tertile of maximum P wave duration.

Figure 1 illustrates the Kaplan-Meier curves for renal end points-free survival in patients subdivided according to the tertiles of P wave dispersion (<83.2, 83.3–96.0, >96.0 ms; log-rank *P*<0.001). The unadjusted HR for tertile 2 *versus* tertile 1 was 1.834 (95% CI, 1.113–3.023, *P* = 0.017) and for tertile 3 *versus* tertile 1 was 2.842 (95% CI, 1.173–4.555, *P*<0.001). Figure 2 illustrates the Kaplan–Meier curves for renal end points-free survival in patients subdivided according to the tertiles of maximum P wave duration (<145.0, 145.0–162.0, >162.0 ms; log-rank *P* = 0.003). The unadjusted HR for tertile 2 *versus* tertile 1 was 1.385 (95% CI, 0.861–2.226, *P* = 0.179) and for tertile 3 *versus* tertile 1 was 2.115 (95% CI, 1.350–3.314, *P* = 0.001).

The addition of P wave dispersion and maximum P wave duration to a basic model (including age, sex, DM, hypertension, coronary artery disease, cerebrovascular disease, LVH, systolic and diastolic blood pressure, BMI, albumin, fasting glucose, log triglycerides, total cholesterol, hemoglobin, baseline eGFR, calcium-phosphorus product, uric acid, and proteinuria) significantly improved the prediction value for renal end points (difference in the –2 log likelihood value: 13.531, *P*<0.001 and 6.335, *P* = 0.012, respectively).

Patients with CKD stage 5 (n = 131) were at an extremely high risk of reaching end-stage renal disease in a very short term. Therefore, we performed subgroup analysis after excluding those patients. The results were similar, i.e., patients with increased P wave dispersion (HR, 1.060; 95% CI, 1.031–1.089; *P*<0.001) and maximum P wave duration (HR, 1.048; 95% CI, 1.022–1.074; *P*<0.001) were significantly associated with progression to renal end points after multiple adjustments for demographic, clinical, and biochemical factors.

### Risk of faster renal function decline

Table 3 shows the determinants of the eGFR slope. In the first multivariate analysis, P wave dispersion was negatively associated with the eGFR slope after adjustment for age and sex (unstandardized coefficient β = –0.020, *P* = 0.015) and, further adjustment for smoking, DM, hypertension, coronary artery disease, cerebrovascular disease, LVH, systolic and diastolic blood pressure, BMI, albumin, fasting glucose, log triglycerides, total cholesterol, hemoglobin, baseline eGFR, calcium-phosphorus product, uric acid, and proteinuria (unstandardized coefficient β = –0.016, *P* = 0.037). In the second multivariate analysis, maximum P wave duration was also negatively associated with the eGFR slope (unstandardized coefficient β = –0.014, *P* = 0.040) after adjustment for demographic, clinical, and biochemical factors.

**Table 3:** Determinants of the eGFR slope.

	P wave dispersion		Maximum P wave duration	
	unstandardized coefficient β (95% CI)	*P*	unstandardized coefficient β (95% CI)	*P*
Unadjusted	–0.020 (–0.036, –0.004)	0.017	–0.021 (–0.036, –0.007)	0.003
Age and sex adjusted	–0.020 (–0.036, –0.004)	0.015	–0.021 (–0.035, –0.006)	0.005
Multivariate adjusted	–0.016 (–0.031, 0)	0.037	–0.014 (–0.028, 0)	0.040

Abbreviations: eGFR, estimated glomerular filtration rate.

Values expressed as unstandardized coefficient β and 95% confidence interval (CI).

Multivariate model: adjusted for age, sex, smoking history, diabetes mellitus, hypertension, and coronary artery disease, cerebrovascular disease, left ventricular hypertrophy, systolic and diastolic blood pressure, body mass index, albumin, fasting glucose, log triglycerides, total cholesterol, hemoglobin, baseline eGFR, calcium-phosphorus product, uric acid, and proteinuria.

The addition of P wave dispersion and maximum P wave duration to a basic model (including age, sex, DM, hypertension, coronary artery disease, cerebrovascular disease, LVH, systolic and diastolic blood pressure, BMI, albumin, fasting glucose, log triglycerides, total cholesterol, hemoglobin, baseline eGFR, calcium-phosphorus product, uric acid, and proteinuria) significantly improved the prediction value for renal function decline (difference in the adjusted R square value: 0.01, *P* = 0.037 and 0.009, *P* = 0.040, respectively).

### Determinants of P wave dispersion and maximum P wave duration

In univariate analysis, P wave dispersion had a significant positive correlation with DM (unstandardized coefficient β = 4.366, *P* = 0.020). In addition, maximum P wave duration had a significant positive correlation with DM, fasting glucose, log triglycerides, and proteinuria. In multivariate analysis, maximum P wave duration was independently correlated with DM (unstandardized coefficient β = 5.635, *P* = 0.015).

### Reproducibility of P wave measurements

The intraobserver mean percent errors for P wave dispersion and maximum P wave duration measurements were 8.6±7.8% and 4.0±3.0%, respectively. The interobserver mean percent errors for P wave dispersion and maximum P wave duration measurements were 10.4±7.3% and 5.0±3.5%, respectively.

## Discussion

In the present study, we evaluated the association of P wave parameters with kidney disease progression to dialysis or death and the rate of renal function decline in patients with CKD stages 3–5. Increased P wave dispersion and maximum P wave duration were associated with progression to dialysis or death and faster renal function decline. Furthermore, the addition of P wave dispersion and maximum P wave duration to a model of clinical features significantly improved the prediction value for progression to dialysis or death and faster renal function decline.

Paoletti et al. [26] studied the role of LVH in the prediction of progression to dialysis in 144 patients with CKD stages 3–4. They found that increased left ventricular mass index was independently associated with CKD progression to dialysis and with combined end points of dialysis or death. Shlipak et al. [27] demonstrated that decreased left ventricular systolic function was an independent predictor of rapid renal function decline (defined as an annual eGFR loss >3 mL/min/1.73 m^2^) in the elderly. We recently reported that concentric LVH, increased left atrial diameter, and decreased left ventricular ejection fraction were associated with adverse renal outcomes [5], [6]. This suggested that patients with structural or functional abnormalities of the heart might have a faster renal function decline and worse renal outcomes. The mechanisms of faster renal function decline in patients with cardiac abnormalities are multi-factorial, including chronic renal hypoperfusion, subclinical inflammation, endothelial dysfunction, accelerated atherosclerosis, increased renal vascular resistance, systemic neurohormonal factors, pharmacotherapies, and anemia [28], [29]. Previous studies have reported that increased P wave dispersion and maximum P wave duration were correlated with left atrial enlargement, LVH, and left ventricular systolic and diastolic dysfunction [12], [16], [17]. Furthermore, we have previously reported that increased P wave dispersion and maximum P wave duration were associated with progression to the renal end point of ≧ 25% decline in eGFR in 166 patients with a mean eGFR of 57.0±17.2 mL/min/1.73 m^2^ [17]. The present study also revealed that increased P wave dispersion and maximum P wave duration were associated with progression to dialysis or death and faster renal function decline in CKD stages 3–5 patients with a mean eGFR of 26.1±14.2 mL/min/1.73 m^2^.

Diabetic nephropathy is one of the major complications of DM and one of the major causes of renal replacement therapy [30]. Cardiovascular disease is the leading cause of morbidity and mortality in patients with diabetic nephropathy [31]. This amplified cardiovascular risk is partly attributable to increased traditional risk factors among diabetic patients, along with the non-traditional risk factors among CKD patients, such as proteinuria, fluid retention, anemia, oxidative stress, and the presence of a chronic inflammatory state [31]–[33]. There are a number of hemodynamic and metabolic disturbances that affect the structure and function of the heart in patients with diabetic nephropathy. The major factors that contribute to further heart failure in diabetic patients include cardiac microangiopathy, neuropathy of the cardiac autonomic nervous system, disturbed metabolism, and fatty degeneration of the myocardium [34]. These patients were reported to have a high prevalence of decreased left ventricular systolic function and increased left ventricular mass index due to pressure and volume overload [35]–[37]. Our results demonstrated a significant association between P wave parameters and DM, which might be partially explained by ischemic or non-ischemic diabetic cardiomyopathy.

Our study had some limitations. The number and interval of serum creatinine measurements varied in each patient. We therefore excluded patients with less than three eGFR measurements during the follow-up period and those patients with a follow-up period of <3 months, in order to decrease the chance of not completely observing a change in renal function. In addition, P wave parameters cannot completely replace echocardiography because they do not provide a comprehensive examination of the heart. However, they can provide a simple and inexpensive method for detecting patients at risk of left atrial enlargement and left ventricular diastolic and systolic dysfunction.

In conclusion, our results demonstrated that increases in P wave dispersion and maximum P wave duration were independently associated with progression to the renal end points of dialysis or death and faster renal function decline in patients with CKD stages 3–5. Beyond conventional clinical features, measurement of P wave dispersion and maximum P wave duration offers an extra benefit in predicting the renal end points of dialysis or death and faster renal function decline. Screening CKD patients on the basis of P wave dispersion and maximum P wave duration, measured by 12-lead ECG, may help identify patients at high risk for worse renal outcomes.
